# PIWI‐interacting RNA MIABEPIR regulates cerebral endothelial cell function via DAPK2 pathway in offspring following maternal immune activation

**DOI:** 10.1002/ctm2.70260

**Published:** 2025-02-25

**Authors:** Shan‐Shan Li, Miao Guo, Yao Long, Yuang Cai, Ying Zhao, Shaoyuan Huang, Houzhi Yang, Yonggang Fan, Xu Chen, Xin Jin

**Affiliations:** ^1^ School of Medicine Nankai University Tianjin China; ^2^ Tianjin Medical University Tianjin China; ^3^ Tianjin Central Hospital of Gynecology Obstetrics Tianjin China; ^4^ Tianjin Key Laboratory of Human Development and Reproductive Regulation Tianjin China

**Keywords:** autophagy, blood–brain barrier, brain microvascular endothelial cell, maternal immune activation, PIWI‐interacting RNA

## Abstract

Maternal immune activation (MIA) is recognised as a risk factor in the neurodevelopmental disorders. However, the precise molecular pathways through which MIA disrupts neurovascular function remain largely unexplored. Here, we identify a novel MIA‐associated brain endothelial piRNA (MIABEPIR) involved in regulating BMEC function and BBB integrity. RNA microarray analysis of foetal brain tissue from MIA‐exposed mice revealed significant changes in piRNA expression, including a marked upregulation of MIABEPIR upregulated piRNAs. Immunofluorescence and FISH confirmed that MIABEPIR is localised in the microvascular endothelial cells of the brain. MIABEPIR overexpression enhances BMEC proliferation and angiogenesis but disrupts BBB integrity. In vivo, intracranial administration of lentiviral MIABEPIR in foetal mice resulted in marked BBB disruption. Mechanistically, we identified DAPK2 as a downstream target of MIABEPIR, leading to its downregulation. This suppression of DAPK2 inhibits autophagy in BMECs, suggesting that MIABEPIR modulates endothelial cell autophagy through the DAPK2 pathway. Our findings reveal a novel piRNA‐mediated regulatory mechanism in neurovascular function during MIA and highlight MIABEPIR's role in MIA‐induced neurodevelopmental abnormalities. Targeting the MIABEPIR‐DAPK2 axis represents a potential therapeutic strategy for addressing neurovascular dysfunction in neurodevelopmental disorders associated with maternal immune stress.

## Graphical abstract

MIABEPIR, a brain endothelial piRNA, as a key regulator of neurovascular dysfunction in maternal immune activation. MIABEPIR modulates BBB integrity and endothelial autophagy through the Dapk2 pathway, highlighting a potential therapeutic target for neurodevelopmental disorders.

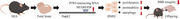

## 1 INTRODUCTION

Maternal immune activation (MIA) during gestation is a widely acknowledged environmental influence that affects fetal brain maturation and elevates the likelihood of neurodevelopmental disorders.^1^, ^2^, ^3^ MIA induces a pro‐inflammatory state that interferes with fetal brain development, leading to persistent cognitive impairments and behavioral abnormalities in the offspring.^4^, ^5^, ^6^ Evidence suggests that disruptions in the BBB contribute significantly to the neurodevelopmental impairments associated with MIA.^7^, ^8^, ^9^ However, the detailed molecular mechanisms driving these effects in the context of MIA remain insufficiently understood.

piRNAs are primarily known for their role in regulating germline development by silencing transposable elements and maintaining genomic stability. Recent research has expanded their functional significance to somatic tissues, where they are involved in gene regulation, epigenetic control, and post‐transcriptional modifications.^10^, ^11^ piRNAs are now recognized for their roles in the brain, influencing neurogenesis, and synaptic plasticity, and potentially contributing to brain aging and neurodegenerative diseases.^12^, ^13^, ^14^ Moreover, piRNAs have been implicated in immune regulation, including autoimmune diseases, immunodeficiency and infections.^15^ Given their roles in maintaining genomic stability and regulating inflammation—two processes implicated in MIA—piRNAs are likely relevant to MIA‐associated neurodevelopmental disorders. However, the specific functions of piRNAs in MIA‐induced neurodevelopmental disorders remain unexplored.

We investigated the dysregulation of piRNAs in fetal brain tissue following MIA. We identified MIABEPIR as a key piRNA that is significantly upregulated in the microvascular endothelial cells of MIA‐exposed fetal brains. Our study demonstrates that MIABEPIR modulates autophagy in BMECs via its downstream target, DAPK2. Our results show the important role of piRNAs in regulating endothelial and neurodevelopmental processes and suggest potential therapeutic targets for mitigating the effects of maternal immune activation on fetal brain development.

## 2 MATERIALS AND METHODS

### 2.1 Animals

8 to 10 week‐old C57BL/6J mice (Sibeifu) were used to establish the MIA model. Mice were grouped by sex, with three to five animals per cage, and maintained under standardized laboratory conditions. After the acclimatization period, breeding pairs were established by co‐housing one male with two females (1:2 ratio). Mating was initiated at the onset of the dark cycle by introducing a single male into a cage with females. Males remained with the females overnight, and the presence of a vaginal plug was checked the following morning. Males were then removed, and females were examined for vaginal plug formation, with the day of detection designated as embryonic day 0.5 (E0.5). Pregnancy was confirmed by a weight gain of ≥2 g by embryonic day E9.5.

To induce maternal immune activation (MIA), polyinosinic‐polycytidylic acid (Poly(I:C)) (APExBIO, B5551) was dissolved in 0.9% NaCl and administered via intraperitoneal injection at embryonic day 9.5 (E9.5) at a dose of 20 mg/kg with an injection volume of 5 mL/kg. The control group received an intraperitoneal injection of 0.9% NaCl at an equivalent volume. All experimental procedures adhered to the guidelines of Nankai University's Animal Care and Use Committee (Approval No. 2023‐000408).

### 2.2 piRNA expression array analysis

piRNA expression profiling was conducted using the Arraystar MM9 piRNA array (Aksomics Inc.). Brain tissue samples were collected from five MIA‐exposed E10.5 fetal mice and five control E10.5 fetal mice. The samples were immediately preserved in Trizol (Invitrogen, 15596026) and stored at –80°C. Mouse PIWI‐interacting RNAs (piRNAs) retrieved from the NCBI database were aligned to the MM9 genome assembly using UCSC BLAT, resulting in the design of over 43,000 piRNA probes (Kangcheng Biological Co., Ltd). A total of 1 µg of RNA per sample was labelled at the 3′ end with Cy3 fluorescent dye through T4 RNA ligase‐mediated ligation. The RNA labeling process included incubation with CIP buffer and calf intestinal phosphatase (CIP, Exiqon) at 37°C for 30 min, followed by an enzyme inactivation through heat treatment at 95°C for 5 min. Next, labeling buffer, Cy3 fluorescent dye, DMSO, and the labeling enzyme were introduced into the reaction mixture, which was then incubated at 16°C for 1 h. The enzymatic reaction was halted by heat inactivation at 65°C for 15 min. The Cy3‐labelled RNA samples were subsequently hybridized onto the Arraystar Mouse piRNA Array within Agilent SureHyb Hybridization Chambers at 65°C for 17 h. Signal intensities were extracted using Agilent Feature Extraction software, and quantile normalization was conducted using GeneSpring GX v12.1. Differentially expressed piRNAs were identified based on fold‐change threshold criteria.

### 2.3 Cell culture and treatments

bEnd.3 cells (Procell Life Science & Technology) were cultured in high‐glucose DMEM (Solarbio, 12100) supplemented with 10% FBS (ExCell, FCS500) and 1% penicillin‐streptomycin (Gibco, 10378016) at 37°C with 5% CO_2_.

To alter MIABEPIR expression, cells were transfected with MIABEPIR mimics (100 nM) or inhibitors (GenePharma) (100 nM) via Lipofectamine 2000 (Invitrogen, 11668‐019). Non‐targeting negative control (NC) (100 nM) transfections were included as experimental control. To simulate inflammatory conditions, bEnd.3 cells were treated with Poly(I:C) (10 µg/mL, APExBIO) or the following pro‐inflammatory cytokines: IL‐6 (50 ng/mL); IL‐1β (50 ng/mL); TNF‐α (50 ng/mL); CCL‐2 (50 ng/mL). Cells were incubated with Poly(I:C) or cytokines for 24 h before further analysis. Untreated cells served as controls.

### 2.4 CCK‐8 cell viability

Cell viability was assessed with the Cell Counting Kit‐8 (CCK‐8, TransGen, FC101) following the protocol provided by the manufacturer. Cells were seeded at a density of 2 × 10^3^ cells per well in 96‐well plates and incubated for 24 h. Twenty‐four hours post‐transfection, cells were washed three times with phosphate‐buffered saline (PBS), and 100 µL of fresh culture medium containing 10% FBS and 1% penicillin‐streptomycin, followed by the addition of 10 µL of CCK‐8 solution. The plates were then incubated at 37°C in a 5% CO₂ atmosphere for 1 h. To evaluate cell viability, absorbance at 450 nm was measured using a microplate reader.

### 2.5 Flow cytometry analysis

bEnd.3 cells were harvested using 0.25% Trypsin‐EDTA (Gibco, 25200072) and collected by centrifugation. The cell pellet was washed with 1 mL of pre‐chilled 1× PBS, centrifuged, and resuspended in 1 mL of pre‐chilled 70% ethanol for overnight fixation at 4°C. The fixed cells were centrifuged and washed with pre‐chilled 1× PBS. Each sample was stained with 500 µL of propidium iodide (PI) solution (Beyotime, C1052) and incubated at 37°C for 30 min. The labelled cells were examined for cell cycle distribution utilizing a flow cytometer, and the acquired data were processed to assess the proportion of cells in different cell cycle phases.

### 2.6 Tube‐like formation assay

Matrigel (Corning, 356255) was thawed overnight at 4°C. Both 200 µL pipette tips and a 96‐well plate were pre‐cooled. A total of 70 µL of Matrigel was dispensed into each well of a pre‐chilled plate and allowed to solidify at 37°C for 30 min. bEnd.3 were then rinsed three times with D‐PBS (Solarbio, D1040) and subsequently incubated with 2 mL of Accutase (Invitrogen, A11105‐01) at 37°C for 2 min to facilitate cell detachment. To terminate the reaction, complete DMEM was added, and the cells were subsequently centrifuged at 1,000 rpm for 5 min to facilitate pellet formation. The cell pellet was resuspended in complete DMEM supplemented with 100 ng/mL VEGF‐A (Solarbio, P00145), and 100 µL of this cell suspension was seeded into each well containing solidified Matrigel. Images of tube formation were captured after 8 h to assess endothelial tube formation and branching.

### 2.7 Cell permeability assay

Transwell inserts (Corning, 3413) were pre‐treated with 15 µg/mL collagen I (Solarbio, C8062) and 30 µg/mL fibronectin (Solarbio, F8180) for 10 min to facilitate cell adhesion and extracellular matrix support. The BMECs were seeded into the coated chambers. Twenty‐four hours post‐transfection, cell permeability was assessed by adding 4.4 kDa TRITC‐dextran (Sigma, T1037) or 70 kDa FITC‐dextran (Sigma, 46945) at 2 mg/mL to the upper chamber. Samples of 20 µL were taken from the lower chamber at 1‐h intervals for up to 7 h, and supplemented with 80 µL of 1× PBS for absorbance measurements. Absorbance was measured using a microplate reader, and dextran concentration was calculated based on a standard curve.

### 2.8 RNA sequencing analysis

Total RNA was isolated from foetal brain tissue using TRIzol Reagent (Invitrogen Life Technologies). RNA purity was evaluated using the kaiaoK5500® Spectrophotometer (Kaiao), while RNA integrity and concentration were assessed with the RNA Nano 6000 Assay Kit and the Agilent Bioanalyzer 2100 system. For library construction, 3 µg of total RNA per sample was processed using the RNA Library Prep Kit for Illumina. Sequencing was performed on the Illumina HiSeq platform at Shanghai Personal Biotechnology Co., Ltd. The high‐quality reads were then aligned to the mouse reference genome (GRCm39) using HISAT2. Transcript assembly was conducted with StringTie, and gffcompare was employed to merge individual transcriptomes into a comprehensive dataset. To identify differentially expressed genes (DEGs), analysis was performed using DESeq2, applying the following significance criteria: *p*‐value < 0.05, log_2_fold‐change ≥ 1 for upregulated genes, and log_2_fold‐change ≤ –1 for downregulated genes.

To functionally characterize the DEGs, GSEA was conducted using the Enrichr platform (https://maayanlab.cloud/Enrichr/). The analysis was performed across the following Gene Ontology (GO) categories: Biological Processes, Cellular Components, and Molecular Functions. Enrichment results were ranked based on *p*‐values to identify significantly enriched terms.

### 2.9 Luciferase reporter assay

The dual‐luciferase reporter assay was performed to investigate the interaction between piRNA MIABEPIR and the DAPK2 3′‐UTR. We utilized the pSI‐Check2 vector (Promega, C8021) for the assay. The 3′‐UTR of DAPK2 was cloned downstream of the Renilla luciferase gene using the XhoI and NotI restriction sites. This placement allows Renilla luciferase activity to be modulated in response to piRNA MIABEPIR binding to the DAPK2 3′‐UTR. Trans5α chemically competent cells (TransGen, CD201) were used for plasmid propagation. The constructed wild‐type and mutant psiCHECK‐2 reporter vectors were transfected into bEnd.3 cells using Lipofectamine 2000 (Invitrogen, 11668‐019) according to the manufacturer's protocol. Luciferase activity was measured using the Dual‐Luciferase Reporter Assay System (Promega, E1910). The firefly luciferase signal, serving as an internal control, was detected with the firefly luciferase reaction reagent, while the Renilla luciferase signal, representing the experimental reporter, was quantified using the Renilla luciferase reaction reagent (TransGen, FR201). Luminescence readings were obtained with a BioTek Synergy H1 microplate reader. The Renilla‐to‐firefly luciferase ratio was then computed to evaluate the relative modulation of DAPK2 3′‐UTR activity following piRNA MIABEPIR interaction.

### 2.10 Transmission electron microscopy analysis

bEnd.3 cells were initially fixed in 2.5% glutaraldehyde, then rinsed three times with 0.1 M phosphate buffer. Subsequently, a secondary fixation step was performed using 1% osmium tetroxide at 4°C for 2 h. The specimens underwent gradual dehydration through an ethanol gradient series, followed by embedding in Epon‐Araldite resin and polymerization within moulds to ensure structural preservation. After semi‐thin sectioning to locate regions of interest, ultrathin sections were prepared for microstructural analysis. The sections were counterstained with 3% uranyl acetate and 2.7% lead citrate, then examined under a JEM1400 transmission electron microscope to assess ultrastructural morphology.

### 2.11 In utero injection in the mouse embryonic brain

At E13.5, pre‐pulled glass micropipettes were used for the in‐utero injection procedure. A 2 cm incision was made in the abdominal wall of pregnant mice to expose the embryos. Using a microinjection system, MIABEPIR lentivirus was injected into the lateral ventricles of the foetal brains, while sterile DMEM was injected as a control. Pups were sacrificed at postnatal day 14 (P14), and brain tissues were collected for immunofluorescence staining, qRT‐PCR, and Western blot analysis. At postnatal day 60 (P60), Evans blue permeability assays were performed to assess blood–brain barrier integrity.

### 2.12 Evans blue permeability

At postnatal day 60 (P60), mice injected in utero with MIABEPIR lentivirus into the lateral ventricles received 2% Evans blue dye (2 mL/kg) via tail vein injection. After euthanasia by cervical dislocation, PBS was perfused through the left ventricle to clear the vasculature of blood. Brain tissues were harvested and dissolved in formamide, followed by incubation at 55°C for 24 h to extract the dye. The samples underwent centrifugation, and the supernatant's absorbance was recorded at 620 nm using a microplate spectrophotometer. The Evans blue concentration was determined by referencing a standard calibration curve.

### 2.13 Fluorescence in Situ Hybridization (FISH) and immunofluorescence

Foetal brain tissues from E10.5 mice were collected and fixed overnight at 4°C in 4% PFA. The tissues were then sequentially dehydrated in 10%, 20%, and 30% sucrose solutions until fully saturated and subsequently embedded in OCT compound (SAKURA, 4583). Frozen blocks were stored at −80°C for at least 1 h before sectioning. The cryosections (12 µm thick) were prepared using a cryostat.

To detect MIABEPIR, a Cy3‐labelled MIABEPIR probe (GenePharma) was synthesized and applied to brain sections. Sections were incubated with the probe in a hybridization buffer at 37°C for 12 h following pre‐hybridization treatment. After stringent washing in PBS, select sections underwent additional immunofluorescence staining.

Following FISH, immunofluorescence staining was performed using the following primary antibodies: Rabbit anti‐CD31 (1:100, Abcam), Rabbit anti‐PDGFRβ (1:100, Abcam), Rabbit anti‐Ki67 (1:100, Cell Signalling Technology). The corresponding secondary antibodies included: Coralight594‐conjugated Goat Anti‐Rabbit IgG (1:500, Proteintech), and Coralight488‐conjugated Goat Anti‐Rabbit IgG (1:500, Proteintech). After staining, sections were mounted and imaged using fluorescence microscopy.

### 2.14 Enzyme‐linked immunosorbent assay

The concentrations of IL‐6, IL‐1β, TNF‐α and CCL‐2 in maternal serum were quantified using commercial ELISA kits (R&D Systems). Maternal blood samples were obtained via retro‐orbital bleeding under anaesthesia at E9.5 and allowed to coagulate at room temperature for 30 min. The samples were then centrifuged at 3000 × g for 10 min at 4°C. Absorbance at 450 nm was recorded (BioTek Synergy H1) and optical density values were computed using standard curves generated for each cytokine

### 2.15 qRT‐PCR

Total RNA was isolated from cells and tissues using Trizol reagent (Invitrogen). Reverse transcription into complementary DNA (cDNA) was performed using either universal primers or piRNA‐specific stem‐loop primers in conjunction with reverse transcriptase. The relative expression levels of target RNAs were determined via qPCR using PerfectStart® Green qPCR SuperMix (TransGen, AQ601). U6 and β‐actin were utilized as internal reference genes for normalization. All qRT‐PCR experiments were conducted in triplicate, and relative gene expression was analysed using the 2^−ΔΔCt^ method to ensure accuracy and reproducibility.

### 2.16 Western blotting

The protein was extracted from cells using radio immunoprecipitation assay buffer (Solarbio, R0010). The concentrations were determined using a bicinchoninic acid (BCA) protein quantification kit (Beyotime, P0010). For each sample, 20 µg of protein was separated via SDS‐polyacrylamide gel electrophoresis (SDS‐PAGE) and transferred onto polyvinylidene difluoride (PVDF) membranes. The membranes were probed with the following primary antibodies: DAPK2 (Rabbit, 1:1000, Cell Signaling Technology), Occludin (Mouse, 1:1000, Proteintech), VE‐Cadherin (Rabbit, 1:1000, Proteintech), Beclin1 (Rabbit, 1:1000, Abcam), and LC‐3 (Rabbit, 1:1000, Proteintech). Following the washing steps, membranes were incubated with horseradish peroxidase (HRP)‐conjugated secondary antibodies, including HRP‐Goat‐anti‐Mouse IgG (1:5000, Proteintech) and HRP‐Goat‐anti‐Rabbit IgG (1:5000, Proteintech). Protein bands were detected using an enhanced chemiluminescence (ECL) detection system and quantified through densitometric analysis. To ensure experimental reliability and accuracy, all assays were conducted in triplicate.

### 2.17 Statistical analysis

All statistical analyses were performed using GraphPad Prism 9.0. Data are presented as mean ± standard error. Two‐group comparisons were conducted using *t*‐tests, while one‐way ANOVA was applied for multiple‐group comparisons, followed by post hoc tests. A *p*‐value < 0.05 was considered statistically significant.

## 3 RESULTS

### 3.1 Dysregulation of piRNA biogenesis genes in MIA foetal brain

**FIGURE 1:**
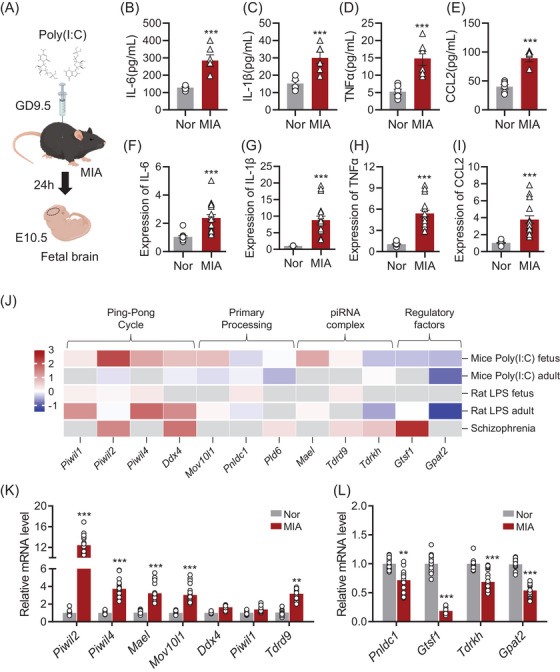
Maternal immune activation (MIA) alters the expression of piRNA biogenesis genes in foetal brain tissue. (A) Schematic illustration of the experimental design involving in utero injection of poly (I:C) at embryonic day 9.5 (E9.5) to induce MIA. ELISA analysis of pro‐inflammatory cytokines, showing significant upregulation of IL‐6 (B), IL‐1β (C), TNF‐α (D), and CCL2 (E) in maternal serum after MIA induction. RT‐qPCR analysis demonstrating significant upregulation of IL‐6 (F), IL‐1β (G), TNF‐α (H), and CCL2 (I) in fetal brain tissue collected at embryonic day 10.5 (E10.5). (J) Heatmap illustrating the differential expression of key genes involved in piRNA biogenesis across publicly available datasets from animal models and human schizophrenia samples. The datasets analysed include: Mice Poly (I:C) foetus (PRJNA602886); Mice Poly (I:C) adult (GSE178403); Rat LPS foetus (GSE34058); Rat LPS adult (GSE185195); Human schizophrenia samples (GSE215985). Core PIWI‐like Proteins: Piwil1 (MIWI), Piwil2 (MILI), and Piwil4 (MIWI2)—essential for primary piRNA processing, participate in the ping‐pong amplification cycle, and regulate transposon repression. piRNA‐processing factors: Mael (Maelstrom), Mov10l1, Ddx4 (Vasa), Tdrd9, Tdrkh, Pld6 (MitoPLD), and Pnldc1 – play critical roles in piRNA precursor cleavage, secondary amplification, and RNA helicase‐mediated unwinding. piRNA Regulatory Proteins: Gtsf1 (Gametocyte‐specific factor 1) and Gpat2 (Glycerol‐3‐phosphate acyltransferase 2)—involved in piRNA maturation, target gene regulation, and downstream functional pathways. RT‐qPCR analysis of piRNA biogenesis pathway genes in foetal brain tissue at E10.5, revealing significant upregulation (K) and downregulation (L) of key genes involved in piRNA biogenesis in response to MIA. Data are shown as mean ± SEM, **p < 0.001, ***p < 0.001 vs. normal (Nor) group.

To induce maternal immune activation (MIA), polyinosinic acid (poly(I:C)) was administered intraperitoneally at gestational day 9.5 (GD9.5). Foetal brain tissue was harvested 24 h later at E10.5 (Figure 1A). Analysis of both maternal serum and foetal brain tissue revealed a significant upregulation of cytokines, including IL‐6 (Figure 1B,F), IL‐1β (Figure 1C,G), TNF‐α (Figure 1D,H), and CCL2 (Figure 1E,I). These findings indicate that MIA triggers a robust inflammatory cascade during pregnancy, extending to the foetal brain environment.

To explore the role of piRNAs in the foetal brain following MIA, we examined the expression of piRNA biogenesis genes using publicly available datasets (Figure 1J). Data mining of these datasets, which included samples from MIA animal models and patients with neurodevelopmental disorders such as schizophrenia, revealed significant dysregulation of key piRNA biogenesis genes. This suggests that MIA‐induced alterations in piRNA biogenesis may contribute to the molecular mechanisms underlying neurodevelopmental disorders. We performed RT‐qPCR on foetal brain tissue from MIA‐exposed offspring mice to assess the expression of piRNA biogenesis genes. Our results confirmed significant dysregulation of critical piRNA biogenesis genes, including *Piwil2*, *Piwil4*, and *Mael*, in the foetal brains of MIA offspring compared to the normal group (Figure 1K,L). The expression patterns identified in our PCR‐based analysis were consistent with RNA‐seq data from foetal tissues (Figure J).

### 3.2 MIABEPIR is significantly upregulated and localized in brain microvascular endothelial cells in MIA‐Exposed foetal brain tissue

**FIGURE 2:**
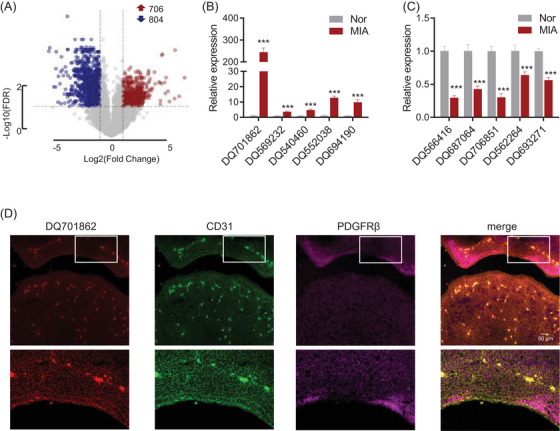
Differential Expression of piRNAs in maternal immune activation (MIA)‐exposed foetal brain tissue and localization of MIABEPIR in microvascular endothelial cells. (A) Volcano plot illustrating the differential expression of piRNAs in MIA‐exposed fetal brain tissue at embryonic day 10.5 (E10.5) using microarray analysis. A total of 1015 piRNAs were upregulated (red dots) and 1041 piRNAs were downregulated (blue dots) in the MIA group compared to controls. RT‐qPCR validation of the five most significantly upregulated (B) and five most significantly downregulated piRNAs (C) identified by the microarray. (D) Immunofluorescence staining of foetal brain tissue sections, showing co‐localization of MIABEPIR (red) with CD31 (green), a marker for microvascular endothelial cells. Merged images confirm the localization of MIABEPIR within endothelial cells of the foetal brain. Data are presented as mean ± SEM, ***p < 0.001 vs. normal (Nor) group.

To investigate the impact of piRNA expression on brain development in MIA‐exposed foetal mice, we performed RNA microarray analysis on E10.5 foetal brain tissue. The results revealed widespread alterations in piRNA expression, with 706 piRNAs upregulated and 804 piRNAs downregulated (FDR < 0.05, ‐logFC > 2) in MIA‐exposed foetal brain tissue compared to normal controls (Figure 2A). To confirm the microarray findings, the five most significantly upregulated and downregulated piRNAs were selected for further validation through RT‐qPCR. Our results confirmed that DQ701862, DQ569232, DQ540460, DQ552038, and DQ694190 were significantly upregulated in the MIA foetal brain compared to controls (Figure 2B), while DQ566416, DQ687064, DQ706851, DQ562264 and DQ693271 were significantly downregulated (Figure 2C). DQ701862 exhibited the most pronounced upregulation, with an approximately 200‐fold increase in expression compared to controls. This marked upregulation, validated across both microarray and RT‐qPCR platforms, identified DQ701862 as the most promising candidate for further functional investigation.

To further investigate the cellular localization of DQ701862, we performed in situ hybridization and immunofluorescence analysis. Our results demonstrated that DQ701862 is predominantly expressed in CD31‐positive cells, a marker of vascular endothelial cells, indicating that DQ701862 is specifically localized to microvascular endothelial cells in the foetal brain (Figure 2D). Given its notable expression in these cells, we propose to name this piRNA DQ701862 as MIA‐associated brain microvascular endothelial piRNA (MIABEPIR) to reflect its potential role in MIA and its specific involvement in brain endothelial cell function. These findings indicate that MIABEPIR may serve as a key regulator of microvascular endothelial cell function during brain development, particularly in response to maternal immune challenges.

### 3.3 MIABEPIR response to inflammatory stimuli and regulation of brain microvascular endothelial cell proliferation

**FIGURE 3:**
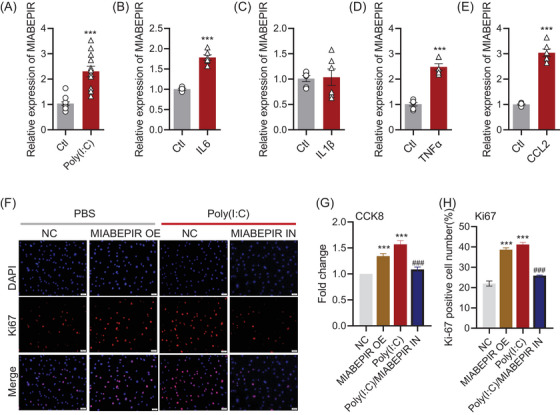
MIABEPIR promotes brain microvascular endothelial cell (BMEC) proliferation under inflammatory conditions. (A–E) qRT‐PCR analysis showing MIABEPIR expression levels in bEnd.3 cells treated with 10 µg/mL poly(I:C) (A), 50 ng/mL IL‐6 (B), 50 ng/mL IL‐1β (C), 50 ng/mL TNF‐α (D), and 50 ng/mL CCL2 (E), revealing significant upregulation of MIABEPIR in response to these pro‐inflammatory stimuli compared to controls. (F) Immunofluorescence images of Ki67‐positive nuclei (red) in control, MIABEPIR overexpression, Poly(I:C) treatment, and MIABEPIR inhibition groups. DAPI staining (blue) was used to visualize the nuclei. (G) Quantification of cell proliferation via CCK‐8 assay in control, MIABEPIR overexpression, Poly (I:C) treatment, and MIABEPIR inhibition groups. (H) Quantification of Ki67‐positive cells from immunofluorescence images. Data are presented as mean ± SEM, ***p < 0.001 vs. normal (Nor) or negative control (NC) group; ###*p* < 0.001 vs. Poly (I:C) group.

To investigate the regulatory role of MIABEPIR in brain microvascular endothelial cells (BMECs) under inflammatory conditions, we treated bEnd.3 cells with Poly (I:C) and various pro‐inflammatory cytokines, including IL6, IL1β, TNF‐α and CCL‐2. qRT‐PCR analysis revealed that MIABEPIR expression was significantly upregulated in response to Poly (I:C) (Figure 3A), IL‐6 (Figure 3B), TNF‐α (Figure 3D), and CCL‐2 (Figure 3E).

To assess the functional impact of MIABEPIR on BMECs, we transfected bEnd.3 cells with MIABEPIR mimics and inhibitors. Compared to the negative control (NC) group, MIABEPIR overexpression through mimics, as well as Poly(I:C) treatment, significantly enhanced BMEC proliferation, as shown by CCK‐8 (Figure 3G) and Ki67 staining (Figure 3F,H). Conversely, inhibition of MIABEPIR under Poly (I:C) conditions reversed the proliferation of bEnd.3 cells induced by Poly (I:C) (Figure 3G,H).

**FIGURE 4:**
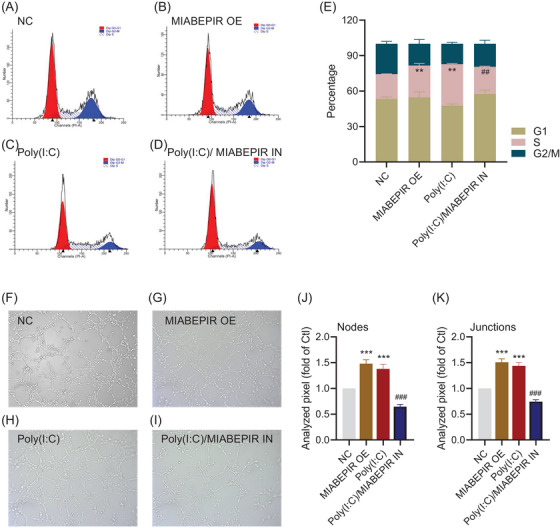
MIABEPIR regulates cell cycle progression and tube formation in brain microvascular endothelial cells (BMECs). Cell cycle analysis by flow cytometry in control (Ctl) (A), MIABEPIR overexpression (OE) (B), Poly(I:C) (C), and Poly(I) + MIABEPIR inhibition (IN) (D) groups. The bar chart (E) represents the quantification of the percentage of cells in the G1, S, and G2/M phases of the cell cycle. (F) Representative images of tube formation assay in control (F), MIABEPIR OE (G), Poly (I:C) (H), and Poly(I) + MIABEPIR inhibition (I) groups. (G) Quantification of the tube nodes (J) and junctions (H) in each group. Data are presented as mean ± SEM; **p < 0.01, ***p < 0.001 vs. Negative control (NC) group; ##*p* < 0.01, ###p < 0.001 vs. Poly (I:C) group.

Flow cytometry analysis further confirmed the impact of MIABEPIR on cell cycle progression (Figure 4A–D). Overexpression of MIABEPIR or Poly (I:C) treatment reduced the cells in the G1 phase, while increasing the S and M phases, indicating accelerated cell cycle progression (Figure 4E). In contrast, MIABEPIR inhibition under Poly(I:C) conditions reduced the proportion of cells in the S phase. These results suggested that MIABEPIR plays a critical role in driving BMEC proliferation and cell cycle progression during inflammatory stress.

### 3.4 MIABEPIR modulates angiogenesis, BBB permeability, and pericyte recruitment in brain microvascular endothelial cells

To investigate the functional role of MIABEPIR in angiogenesis and BBB permeability under inflammatory conditions, we used an in vitro model to assess endothelial cell tube formation, a key indicator of angiogenesis. MIABEPIR overexpression significantly enhanced tube formation (Figure 4F–I), characterized by a greater number of branching points and longer tube networks compared to control cells (Figure 4J,K). Similarly, cells transfected with MIABEPIR mimics and treated with Poly(I:C) exhibited significantly increased angiogenic activity, as demonstrated by quantification of tube length and branching point analysis. Conversely, inhibition of MIABEPIR under Poly(I:C) conditions reversed these effects, resulting in reduced tube formation, fewer branching points, and shorter networks. These findings highlight MIABEPIR as a critical regulator of angiogenesis during inflammatory stress.

**FIGURE 5:**
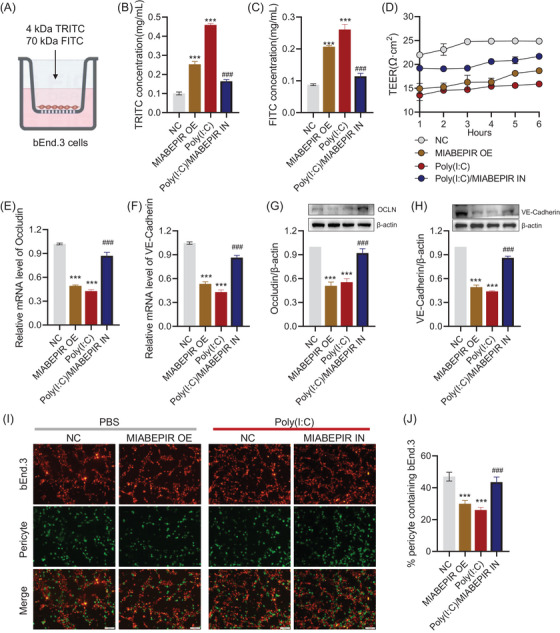
MIABEPIR disrupts blood–brain barrier (BBB) integrity, tight junction function, and pericyte recruitment in brain microvascular endothelial cells (BMECs) under inflammatory conditions. (A) Schematic representation of the transwell permeability assay used to assess BBB integrity in BMECs. Quantification of permeability to 4.4 KDa TRITC‐dextran (B) and 70 KDa FITC‐dextran (C) in BMECs under control, MIABEPIR overexpression (OE), Poly (I:C) treatment, and MIABEPIR inhibition (IN) conditions. (D) Measurement of transendothelial electrical resistance (TEER) under control, MIABEPIR overexpression, Poly(I:C) treatment, and MIABEPIR inhibition conditions. PCR and Western blot analysis and quantification of tight junction proteins occludin (E, G) and VE‐Cadherin (F, H) in BMECs under different conditions. Immunofluorescence images (I) and quantification (J) of pericyte recruitment to BMECs under control, MIABEPIR overexpression, Poly (I:C) treatment, and MIABEPIR inhibition conditions. Data are presented as mean ± SEM; ***p < 0.001 vs. negative control (NC) group; ###*p* < 0.001 vs. Poly (I:C) group.

Next, we assessed the effects of MIABEPIR on blood–brain barrier (BBB) integrity by measuring transendothelial electrical resistance (TEER) and permeability to fluorescently labelled dextran (4 and 70 kDa) using a transwell assay with MBMECs (Figure 5A). Both MIABEPIR overexpression and Poly(I:C) treatment led to significantly reduced TEER values and dextran permeability (Figure 5B–D), indicating weakened barrier function, accompanied by increased dextran permeability. However, inhibition of MIABEPIR improved BBB function under Poly(I:C) conditions, as evidenced by higher TEER values and reduced dextran permeability (Figure 5B–D). RNA and protein expression analyses of key tight junction proteins revealed that MIABEPIR overexpression and Poly(I:C) treatment led to significant downregulation of occludin and claudin‐5 (Figure 5E–H), supporting the notion that MIABEPIR disrupts BBB integrity by weakening tight junctions. In contrast, inhibition of MIABEPIR restored tight junction protein levels under Poly(I:C) treatment (Figure 5E–H), suggesting a protective role for reduced MIABEPIR expression in maintaining BBB function. These findings suggest that MIABEPIR plays a critical role in regulating BBB permeability and potentially contributes to BBB dysfunction under inflammatory conditions.

Pericytes, which are essential for stabilizing endothelial cells and maintaining BBB integrity, were also investigated to determine MIABEPIR's impact on endothelial‐pericyte interactions. Immunofluorescence staining of endothelial‐pericyte co‐cultures revealed that MIABEPIR overexpression, as well as Poly(I:C) treatment, markedly reduced pericyte recruitment to endothelial cells (Figure 5I). Conversely, inhibition of MIABEPIR increased pericyte recruitment, comparable to the control condition (Figure 5J), suggesting that reducing MIABEPIR levels promotes normal pericyte‐endothelial interactions under inflammatory conditions.

### 3.5 Transcriptome analysis reveals endothelial pathways and inflammatory gene signature regulated by MIABEPIR

**FIGURE 6:**
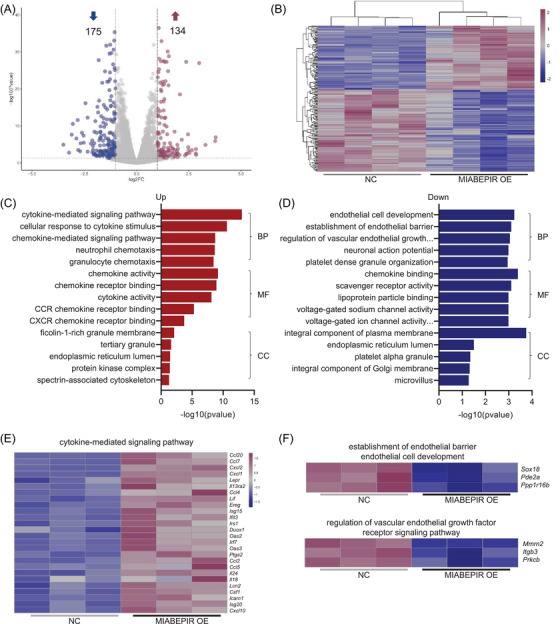
Transcriptome analysis reveals MIABEPIR‐mediated regulation of endothelial pathways and inflammatory gene signatures in response to maternal immune activation (MIA). (A) Volcano plot showing the differentially expressed genes in BMECs overexpressing MIABEPIR compared to controls. A total of 175 genes were downregulated (blue dots), and 134 genes were upregulated (red dots). (B) Heatmap displaying hierarchical clustering of differentially expressed genes (DEGs) between MIABEPIR overexpression and control groups. Upregulated and downregulated genes are shown in red and blue, respectively. Gene ontology (GO) enrichment analysis of upregulated (C) and downregulated (D) genes. (E) Heatmap showing key inflammatory genes upregulated by MIABEPIR overexpression. (F) Heatmap of key downregulation genes involved in the establishment of endothelial barrier and endothelial cell development.

To uncover the molecular mechanisms underlying MIABEPIR's effects on endothelial function, we performed RNA sequencing (RNA‐seq) on MBMECs treated with MIABEPIR mimics. Differential expression analysis identified 175 downregulated and 134 upregulated genes in MIABEPIR‐overexpressing cells compared to controls (Figure 6A,B). Gene enrichment analysis revealed a significant upregulation of genes involved in the cytokine‐mediated signalling pathway (Figure 6C,E), highlighting MIABEPIR's role in promoting inflammatory responses. In contrast, genes essential for the establishment and maintenance of the endothelial barrier were significantly downregulated following MIABEPIR overexpression (Figure 6D,F).

These changes align with the observed impairment of barrier function in MIABEPIR‐overexpressing cells, suggesting that MIABEPIR disrupts critical endothelial pathways responsible for maintaining vascular integrity. These findings provide valuable insight into the molecular landscape regulated by MIABEPIR, emphasizing its dual role in enhancing inflammatory signalling while compromising endothelial barrier function.

### 3.6 MIABEPIR targets DAPK2 in brain microvascular endothelial cells

**FIGURE 7:**
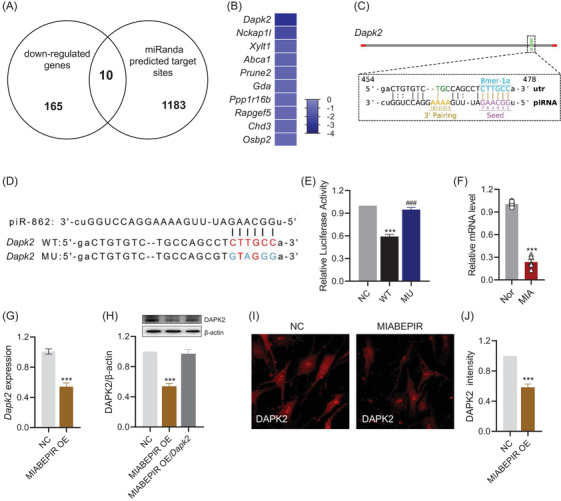
MIABEPIR Targets DAPK2 in endothelial cells. (A) Venn diagram displaying the results of an integrative analysis combining miRanda‐predicted target genes with downregulated genes identified from RNA‐seq data. Target prediction was performed using the miRanda database with the following parameters: minimum free energy ≤ –20 kcal/mol and alignment score ≥ 140. This approach identified 10 potential targets significantly downregulated upon MIABEPIR overexpression, with DAPK2 emerging as the most downregulated gene. (B) Schematic representation of the MIABEPIR binding site in the 3′ untranslated region (3′ UTR) of DAPK2 mRNA, highlighting the predicted 8‐mer seed sequence involved in MIABEPIR binding. (C) Sequence alignment of MIABEPIR with the wild‐type (WT) and mutant (MU) DAPK2 3′ UTRs, showing the mutated nucleotides (in red) used for luciferase reporter assay validation. (D) Luciferase reporter assay demonstrated significant repression of luciferase activity in cells co‐transfected with MIABEPIR and the wild‐type DAPK2 3′ UTR construct, but not in cells transfected with the mutant DAPK2 3′ UTR construct. (E) RT‐qPCR analysis confirms significant downregulation of DAPK2 mRNA expression in the foetal brain following maternal immune activation (MIA). (F–G) PCR and Western blot analyses showed a significant reduction in DAPK2 protein levels in MIABEPIR‐overexpressing cells compared to controls. (H) Immunofluorescence images of DAPK2 protein expression in control and MIABEPIR‐overexpressing endothelial cells confirm decreased DAPK2 expression in the MIABEPIR‐overexpressing cells. (I) Quantification of DAPK2 protein levels from immunofluorescence analysis, showing significant downregulation in MIABEPIR‐overexpressing cells. Data are presented as mean ± SEM. ***p < 0.001 vs. Normal (Nor) or Negative control (NC) group; ###*p* < 0.001 vs. MU group.

To identify downstream targets of MIABEPIR, we performed an integrative analysis combining miRanda‐predicted targets with downregulated genes from our RNA‐seq data (Figure 7A). This analysis revealed 10 potential targets that were significantly downregulated upon MIABEPIR overexpression. Among these candidates, DAPK2 (death‐associated protein kinase 2) emerged as the most significantly downregulated gene (Figure 7B). Further analysis using miRanda predicted a direct binding site for MIABEPIR within the 3′ UTR of DAPK2 mRNA. Specifically, an 8‐mer seed sequence was predicted as a site of interaction between MIABEPIR and the DAPK2 3′ UTR (Figure 7C). To validate this interaction, we conducted a luciferase reporter assay using constructs containing either the WT MU DAPK2 3′ UTR (Figure 7D). Mutation of the seed sequence in the DAPK2‐MU construct was used to assess the specificity of the binding. Luciferase activity in cells co‐transfected with the MIABEPIR mimic and the DAPK2‐WT reporter was significantly reduced compared to controls, indicating that MIABEPIR directly binds to the wild‐type DAPK2 3′ UTR (Figure 7E). However, cells transfected with the DAPK2‐MU (mutant) reporter showed no significant reduction in luciferase activity (Figure 7E), confirming that the predicted seed sequence is essential for the binding of MIABEPIR to the DAPK2 3′ UTR.

To further validate DAPK2 as a downstream target of MIABEPIR, we performed RT‐qPCR (Figure 7G), Western blot (Figure 7H), and immunofluorescence analyses (Figure 7I,J) to examine DAPK2 expression following MIABEPIR overexpression. The results demonstrated a significant reduction in DAPK2 in endothelial cells overexpressing MIABEPIR compared to controls. Additionally, DAPK2 expression was significantly reduced in the foetal brain tissue of MIA‐exposed offspring (Figure 7F). These results confirmed that MIABEPIR negatively regulates DAPK2 expression, establishing DAPK2 as a key downstream target of MIABEPIR.

### 3.7 MIABEPIR regulates endothelial cell autophagy via DAPK2 in brain microvascular endothelial cells

Having identified DAPK2 as a direct downstream target of MIABEPIR, we next examined the functional consequences of MIABEPIR‐mediated DAPK2 regulation in BMECs. Given the established role of DAPK2 in autophagy, we investigated whether MIABEPIR influences endothelial cell autophagy through modulation of DAPK2 expression.

**FIGURE 8:**
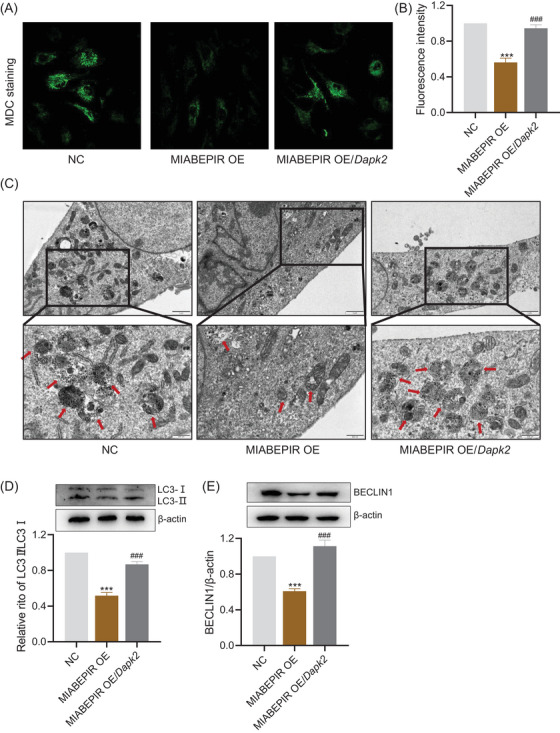
MIABEPIR regulates endothelial cell autophagy via DAPK2 downregulation. (A) Immunofluorescence staining of LC3 in endothelial cells, showing a reduction in LC3 puncta (autophagosomes) in MIABEPIR‐overexpressing cells compared to control cells. (B) Quantification of LC3 puncta, confirming significant suppression of autophagy in MIABEPIR‐overexpressing cells. (C) Transmission electron microscopy (TEM) images displaying autophagosome formation (indicated by red arrows) in control cells, MIABEPIR‐overexpressing cells, and MIABEPIR‐overexpressing cells with DAPK2 rescue. MIABEPIR overexpression markedly reduces autophagosome formation, while DAPK2 overexpression restores autophagosome levels to control levels. (D, E) Western blot analysis of LC3‐II (D) and Beclin‐1 (E) protein levels, showing reduced expression of these autophagy markers in MIABEPIR‐overexpressing cells, with restoration upon DAPK2 overexpression. Data are presented as mean ± SEM. *p* < 0.001 vs. Negative control (NC) group; ###*p* < 0.001 vs. MIABEPIR OE group.

Immunofluorescence staining for LC3, a key marker of autophagosome formation, revealed a significant decrease in LC3 puncta in endothelial cells overexpressing MIABEPIR compared to controls, indicating suppressed autophagy (Figure 8A). Notably, overexpression of DAPK2 rescued this reduction in LC3 puncta, restoring autophagy (Figure 8B). To further confirm the impact of MIABEPIR on autophagy at the ultrastructural level, TEM was used to observe autophagosome formation in endothelial cells (Figure 8C). As shown in the TEM images, control cells exhibited a high number of autophagosomes, consistent with active autophagy. In contrast, MIABEPIR‐overexpressing cells displayed a marked reduction in autophagosome formation, reinforcing the conclusion that MIABEPIR inhibits autophagy (Figure 8C). The decreased number of autophagosomes in MIABEPIR‐overexpressing cells corresponds with the reduction in LC3 puncta. However, when DAPK2 was overexpressed in MIABEPIR‐overexpressing cells, autophagosome formation was significantly restored to levels comparable to the control group, further emphasizing the critical role of DAPK2 in autophagy regulation.

Western blot analysis corroborated these findings, showing reduced expression of autophagic markers LC3‐II (Figure 8D) and Beclin‐1 (Figure 8E) in MIABEPIR‐overexpressing cells compared to controls. Overexpression of DAPK2 restored the expression of these autophagic proteins, confirming that MIABEPIR inhibits autophagy by downregulating DAPK2.

### 3.8 MIABEPIR promotes BBB permeability in offspring following foetal intracranial administration

**FIGURE 9:**
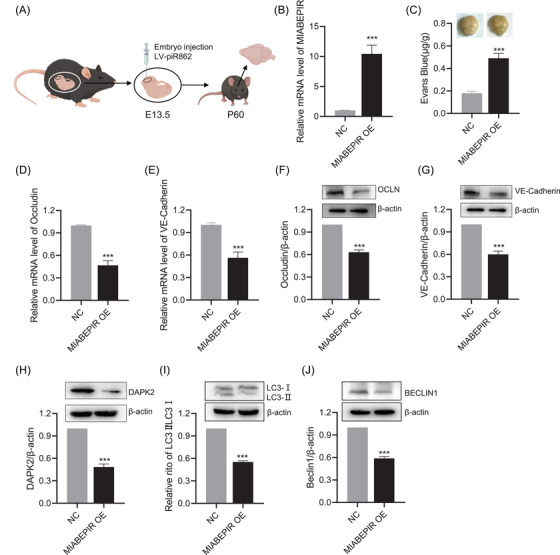
MIABEPIR promotes BBB permeability and autophagic markers and DAPK2 expression in offspring brain following foetal intracranial administration. (A) Schematic representation of foetal intracranial injection of lentiviral (LV)‐MIABEPIR at embryonic day 10 (E10), with analysis performed in offspring at postnatal day 60 (P60). (B) RT‐qPCR analysis confirming significantly elevated MIABEPIR expression in the brains of MIABEPIR‐overexpressing mice compared to controls, indicating successful overexpression. (C) Quantification of Evans blue dye extravasation assay, indicating significantly increased BBB permeability in MIABEPIR‐overexpressing mice compared to controls. (D–G) PCR and Western blot analysis of tight junction proteins occludin (D, F) and VE‐cadherin (E, G), showing reduced expression of these critical BBB proteins in MIABEPIR‐overexpressing mice compared to controls. Western blot analysis of DAPK2 (H), LC3‐II (I), and Beclin‐1 (J) protein levels in brain tissue from MIABEPIR‐overexpressing mice. Data are presented as mean ± SEM. ***p < 0.0001 vs. Negative control (NC) group.

To study the impact of MIABEPIR on BBB function in vivo, we administered lentiviral‐MIABEPIR intracranially to foetal mice at gestational day 13.5 (Figure 9A). In postnatal 60 days offspring, RT‐qPCR analysis confirmed significantly elevated MIABEPIR expression in the brain tissue of MIABEPIR overexpressed mice compared to controls, confirming the successful administration and sustained expression of MIABEPIR (Figure 9B).

To evaluate the impact of MIABEPIR overexpression on BBB integrity, an Evans blue dye extravasation assay was performed. Mice overexpressing MIABEPIR showed a marked increase in Evans blue dye leakage into the brain parenchyma compared to controls, indicating a substantial increase in BBB permeability (Figure 9C). This high level of dye extravasation demonstrates that MIABEPIR compromises BBB integrity, allowing substances to pass through the barrier that would normally be restricted. In addition, RT‐qPCR and Western blot analysis of brain tissues revealed a significant reduction in tight junction proteins, including Occludin (Figure 9D,F) and VE‐Cadherin (Figure 9E,G), in MIABEPIR‐overexpressing mice compared to controls. This reduction in tight junction proteins further underscores MIABEPIR's role in disrupting BBB integrity, leading to increased permeability in vivo.

Additionally, we examined the expression of downstream targets, specifically DAPK2, and autophagic markers. Western blot analysis revealed significant downregulation of DAPK2 (Figure 9H) and a marked reduction in LC3‐II (Figure 9I) and Beclin‐1 protein levels compared to controls (Figure 9J). Together, this in vivo evidence further supports that MIABEPIR regulates autophagy by targeting DAPK2, contributing to disrupted autophagic processes in both endothelial cells and brain tissue.

## 4 DISCUSSION

This study reveals that MIA significantly disrupts piRNA expression patterns in the foetal brain, highlighting their potential role in mediating neurovascular and neurodevelopmental processes. While piRNAs are traditionally associated with transposon silencing and genomic stability in germline cells, emerging evidence indicates their involvement in diverse biological processes, including somatic cell regulation and immune responses.^10^, ^16^, ^17^ Among the altered piRNAs, MIABEPIR emerged as a key player due to its pronounced upregulation in MIA‐exposed foetal brains and its specific localization in brain microvascular endothelial cells (BMECs). This study is the first to establish MIABEPIR's critical role in endothelial cell function and blood–brain barrier (BBB) integrity under inflammatory conditions.

Emerging research on PIWI‐interacting RNAs (piRNAs) in brain function and brain diseases is still in its early stages.^18^, ^19^ It is now clear that piRNAs are involved in gene regulation within the brain, influencing key processes such as neurogenesis^20^ and synaptic plasticity.^21^ Dysregulated piRNA expression has been implicated in several neurological and neurodegenerative diseases, suggesting that piRNAs could serve as biomarkers or therapeutic targets in these conditions. For instance, piRNAs have been shown to regulate neuronal activity and plasticity, with aberrant expression patterns reported in brain diseases.^22^, ^23^, ^24^, ^25^, ^26^, ^27^

piRNAs are well‐established for their role in suppressing transposon mobilization and maintaining genomic integrity in germline cells through interactions with PIWI proteins.^28^ These piRNA‐PIWI complexes are crucial for silencing transposons, protecting the genome during gametogenesis, and modulating chromatin structure.^29^ Our study expands on this understanding by demonstrating that key components of the piRNA biogenesis pathway are significantly altered in response to MIA. This finding suggests that piRNA dysregulation under inflammatory conditions may extend beyond germline cells, potentially playing a role in neurodevelopmental processes and the brain's response to immune stress. We also observed discrepancies between public datasets regarding the expression patterns of PIWI pathway genes. While our PCR‐based analysis focused on foetal mouse tissues, some public datasets derive from adult mouse tissues or different species. These differences likely reflect developmental and interspecies variations in gene regulatory networks, signalling pathways and epigenetic landscapes. These findings indicate the potential influence of piRNA dysregulation on neurodevelopmental processes, highlighting the importance of considering developmental stage and species‐specific contexts in future research.

In our study, we utilized a microarray approach to comprehensively investigate piRNA expression in the foetal brain following MIA. This analysis revealed extensive alterations in the piRNA landscape, with hundreds of piRNAs significantly upregulated or downregulated in response to MIA. Among the most dynamically altered piRNAs, MIABEPIR emerged as a standout candidate due to its pronounced upregulation. piRNAs are well‐established regulators of genomic stability and transposon silencing, primarily in germline cells. However, growing evidence indicates that they may also participate in regulating somatic cell functions, including stress responses and neural development.^30^, ^31^ The dramatic increase in MIABEPIR expression in the foetal brain following poly(I:C) injection aligns with these emerging roles, suggesting it may act as a key mediator of the brain's response to immune stress. Our findings suggest that MIABEPIR is highly responsive to inflammatory stimuli, such as Poly (I:C) and cytokines, reinforcing its involvement in endothelial responses to immune stress. Upregulation of MIABEPIR in endothelial cells was associated with enhanced proliferation and cell cycle progression, indicating a possible role in endothelial repair mechanisms under inflammatory conditions.^32^ Notably, we found that MIABEPIR promotes endothelial tube formation, indicating its involvement in angiogenesis under inflammatory conditions. However, MIABEPIR overexpression also led to increased BBB permeability, as demonstrated by reduced transendothelial electrical resistance and elevated dextran permeability in endothelial monolayers. The downregulation of key tight junction proteins in response to MIABEPIR overexpression supports the result that MIABEPIR compromises BBB integrity by weakening tight junctions. This finding is critical because tight junctions are essential for maintaining the selective permeability of the BBB, and their disruption is a hallmark of neuroinflammatory and neurodevelopmental disorders.^33^, ^34^, ^35^, ^36^, ^37^ Additionally, pericytes play a pivotal role in stabilizing endothelial cells and maintaining BBB function.^38^, ^39^ Our co‐culture experiments revealed that MIABEPIR upregulation impaired pericyte recruitment to endothelial cells, further contributing to BBB dysfunction. The reduced pericyte coverage in the MIABEPIR overexpression group suggests that MIABEPIR‐mediated disruption of endothelial‐pericyte interactions weakens the structural integrity of the BBB. This impaired recruitment may exacerbate vascular permeability, potentially leading to increased neuroinflammation and contributing to neurodevelopmental abnormalities in MIA‐exposed offspring. These findings suggest the important role of MIABEPIR in modulating both angiogenesis and BBB integrity in neurodevelopmental disorders.

We found DAPK2 as a critical downstream target of MIABEPIR, unveiling a novel regulatory pathway that connects MIABEPIR, DAPK2, and autophagic processes in BMECs. DAPK2 is a key regulator of autophagy and apoptosis, a processes vital for maintaining endothelial homeostasis and vascular integrity.^40^, ^41^, ^42^, ^43^ We found that MIABEPIR binds to DAPK2, leading to its downregulation, which has significant implications for blood–brain barrier (BBB) function, particularly under inflammatory conditions.

The downregulation of DAPK2 by MIABEPIR resulted in reduced autophagic flux, evidenced by diminished autophagosome formation and lower levels of key autophagy markers such as LC3‐II and Beclin‐1. Autophagy is essential for clearing damaged organelles and mitigating oxidative stress, processes critical for cellular health. Inhibiting autophagy, as seen with MIABEPIR overexpression, likely disrupts these quality control mechanisms, contributing to endothelial dysfunction. This suppression of autophagy may directly destabilize tight junction complexes, as reduced autophagy has been linked to the accumulation of cellular stress and the degradation of tight junction proteins.^44^, ^45^ These changes can significantly increase BBB permeability, further compromising its integrity.

Beyond its role in autophagy, DAPK2 is also a known mediator of apoptosis.^43^, ^46^ While our data highlight the impact of MIABEPIR on autophagy regulation, the potential influence on DAPK2‐mediated apoptotic pathways remains unexplored in this study. It is plausible that MIABEPIR‐mediated DAPK2 downregulation may suppress apoptotic signalling, potentially promoting endothelial cell survival under inflammatory or stress conditions. However, prolonged suppression of DAPK2 may lead to maladaptive cellular responses, ultimately compromising endothelial function and weakening BBB integrity. These findings suggest that MIABEPIR exerts dual regulatory effects on endothelial cells by modulating autophagy while potentially influencing apoptotic pathways. The observed autophagy suppression appears to promote cell viability while simultaneously disrupting tight junction integrity, thus contributing to BBB dysfunction in the context of maternal immune activation (MIA). In MIA models, inflammatory signals may induce MIABEPIR upregulation, driving the dysregulation of autophagy‐associated pathways and leading to neurovascular impairments.

In vivo, MIABEPIR overexpression resulted in BBB disruption, demonstrated by increased permeability to Evans blue dye, consistent with our in vitro findings. This disruption highlights MIABEPIR's potential involvement in neurodevelopmental disorders characterized by BBB dysfunction. Dysregulation of the MIABEPIR‐DAPK2‐autophagy axis may contribute to these disorders by impairing neurovascular integrity during critical developmental periods. Targeting MIABEPIR offers a promising therapeutic approach. Restoring balanced autophagy and preserving tight junction integrity could mitigate the neurovascular consequences of prenatal inflammation and potentially reduce the risk of long‐term neurodevelopmental impairments.

However, several limitations should be acknowledged: (1) While our experiments offer evidence of MIABEPIR's impact on BBB integrity, we did not utilize MIABEPIR‐deficient models. Future studies employing MIABEPIR‐deficient models could help clarify its functional significance in neurovascular homeostasis and MIA‐induced BBB dysfunction; (2) Our in vivo analyses primarily relied on a MIABEPIR overexpression model, which may not fully capture the complex regulatory interactions and compensatory mechanisms present in a natural inflammatory environment. Future studies should incorporate models with conditional or inducible MIABEPIR knockdown to better understand its context‐dependent roles in BBB integrity; (3) Although we demonstrated that MIABEPIR downregulates DAPK2 and inhibits autophagy, the precise molecular mechanisms governing this interaction remain incompletely understood; (4) Our study primarily focused on its autophagic role. We did not investigate whether MIABEPIR‐mediated DAPK2 downregulation impacts apoptotic signalling in endothelial cells. Investigating the potential crosstalk between autophagic and apoptotic mechanisms will be essential to elucidate the broader implications of MIABEPIR dysregulation.

## 5 CONCLUSIONS

This study shows significant alterations in the piRNA landscape in response to MIA, with MIABEPIR emerging as a critical regulator of endothelial cell function and BBB integrity in the foetal brain. Through its interaction with DAPK2, MIABEPIR inhibits autophagy and disrupts tight junction integrity, leading to increased BBB permeability and endothelial dysfunction. These findings position the MIABEPIR/DAPK2 pathway as a potential therapeutic target for mitigating the long‐term neurodevelopmental impacts of prenatal inflammation. While MIABEPIR was the most dynamically altered piRNA, other differentially expressed piRNAs likely contribute to the broader biological response to MIA. These piRNAs may act directly, reflect compensatory mechanisms, or represent adaptive responses to immune activation. In summary, our current study provides a deeper understanding of the molecular pathways disrupted by prenatal inflammation and opens new avenues for therapeutic interventions aimed at protecting foetal brain development from the adverse effects of MIA.

## AUTHOR CONTRIBUTIONS

*Acquisition of data*: Miao Guo, Shan‐Shan Li and Yao Long. *Data analysis*: Miao Guo, Shan‐Shan Li, Yao Long, Yuang Cai, Ying Zhao, Shaoyuan Huang, Houzhi Yang, Yonggang Fan and Xu Chen. *Writing of the manuscript*: Shan‐Shan Li and Xin Jin. *Review and/or revision of the manuscript*: Shan‐Shan Li and Xin Jin. *Conception and design*: Shan‐Shan Li and Xin Jin. *Study supervision*: Xin Jin.

## CONFLICT OF INTEREST STATEMENT

The authors declare no conflicts of interest.

## ETHICS STATEMENT

All experimental protocols were carefully designed to minimize any potential distress or discomfort to the animals. These protocols were approved by the Nankai University Animal Care and Use Committee (2023‐000408), following institutional guidelines for the ethical treatment of animals in research.

## Data Availability

The data in support of the results are available from the corresponding author on reasonable request.
